# Depletion of Microglia in an *Ex Vivo* Brain Slice Culture Model of West Nile Virus Infection Leads to Increased Viral Titers and Cell Death

**DOI:** 10.1128/spectrum.00685-22

**Published:** 2022-04-12

**Authors:** Sarah Stonedahl, Jennifer Smith Leser, Penny Clarke, Kenneth L. Tyler

**Affiliations:** a Department of Immunology and Microbiology, University of Colorado, Aurora, Colorado, USA; b Department of Neurology, University of Colorado, Aurora, Colorado, USA; c Division of Infectious Disease, Department of Medicine, University of Colorado, Aurora, Colorado, USA; d Denver Veteran Affairs Medical Center, Aurora, Colorado, USA; Thomas Jefferson University

**Keywords:** West Nile virus, microglia, viral pathogenesis

## Abstract

West Nile virus (WNV) is a major cause of viral encephalitis in the United States. WNV infection of the brain leads to neuroinflammation characterized by activation of microglia, the resident phagocytic cells of the central nervous system (CNS). In this study, depletion of CNS microglia using the CSF1R antagonist PLX5622 increased the viral load in the brain and decreased the survival of mice infected with WNV (strain TX02). PLX5622 was also used in *ex vivo* brain slice cultures (BSCs) to investigate the role of intrinsic neuroinflammatory responses during WNV infection. PLX5622 effectively depleted microglia (>90% depletion) from BSCs resulting in increased viral titers (3 to 4-fold increase in PLX5622-treated samples) and enhanced virus-induced caspase 3 activity and cell death. Microglia depletion did not result in widespread alterations in cytokine and chemokine production in either uninfected or WNV infected BSCs. The results of this study demonstrated how microglia contribute to limiting viral growth and preventing cell death in WNV infected BSCs but were not required for the cytokine/chemokine response to WNV infection. This study highlighted the importance of microglia in the protection from neuroinvasive WNV infection and demonstrated that microglia responses were independent of WNV-induced peripheral immune responses.

**IMPORTANCE** WNV infections of the CNS are rare but can have devastating long-term effects. There are currently no vaccines or specific antiviral treatments, so a better understanding of the pathogenesis and immune response to this virus is crucial. Previous studies have shown microglia to be important for protection from WNV, but more work is needed to fully comprehend the impact these cells have on neuroinvasive WNV infections. This study used PLX5622 to eliminate microglia in an *ex vivo* brain slice culture (BSC) model to investigate the role of microglia during a WNV infection. The use of BSCs provided a system in which immune responses innate to the CNS could be studied without interference from peripheral immunity. This study will allow for a better understanding of the complex nature of microglia during viral infections and will likely impact the development of new therapeutics that target microglia.

## INTRODUCTION

West Nile Virus (WNV) is a neurotropic, mosquito-borne, single-stranded RNA virus in the *Flaviviridae* family. In nature, the virus is maintained in an enzootic cycle between mosquitos (*Culex* species) and birds. Humans can become infected, along with some other mammals, but are considered dead-end hosts because the subsequent viremia is not sufficient to transmit the virus (1). West Nile virus is the leading cause of epidemic viral encephalitis in the United States and has caused over 25,000 cases of neuroinvasive disease in the United States since its introduction into the US in 1999 (1). Most human infections are asymptomatic (∼80%) while some result in West Nile fever, an acute illness characterized by a nonspecific viral prodrome of fever, headaches, muscle aches, nausea, fatigue, and lymphadenopathy. Less than 1% of human infections result in severe neurological manifestations, including encephalitis, meningitis, or myelitis. Patients with neuroinvasive WNV disease have a mortality rate of 10%, and approximately 50% of survivors have long-lasting neurological effects, including motor impairment or cognitive sequelae (1, 2).

Following systemic infection, WNV gains access to the CNS by crossing the blood-brain barrier (BBB). The pathogenesis of viral entry into the CNS is not yet fully understood; however, there have been several proposed mechanisms, including a cytokine-mediated increase in permeability of the BBB, hematogenous entry by infection of infiltrating immune cells (Trojan Horse model), and retrograde axonal transport from the periphery (2, 3). Following the invasion of the CNS, WNV directly infects neurons causing neuronal cell death by caspase 3-dependent apoptosis (4, 5). CNS tissue injury caused by neuroinvasive WNV infections likely results from both neuronal cell death and neuroinflammation (5). This neuroinflammation is characterized by activation of microglia and astrocytes, production of inflammatory cytokines, breakdown of the BBB, and infiltration of peripheral immune cells (6).

Microglia are of myeloid origin and arise from the yolk sac along with other monocytes. They migrate to the CNS during early development (7–9). Microglia are the resident mononuclear phagocytic cells of the CNS and play multiple roles within the CNS such as immune defense, maintenance of homeostasis, and synaptic pruning during early development. They persistently monitor the CNS for signs of danger and injury, including neuronal damage and the presence of pathogens (10–12). Current literature suggests microglia become activated during WNV infection of the CNS in both humans (13, 14) and mice (15–17). Activated microglia undergo morphological changes and display increased motility, proliferation, and production of inflammatory cytokines (18–22). In neurodegenerative conditions, microglia are thought to contribute to pathogenicity through overactivation (21, 23, 24). However, in the case of viral encephalitis, it remains unclear whether microglia are beneficial or detrimental to disease outcomes as the inflammatory response may have the beneficial effect of inhibiting viral replication and spread or the deleterious effect of exacerbating neuronal death.

Microglia recognize WNV particles through Toll-like receptor 3 (TLR3) signaling and initiate an innate immune response against the viral infection involving increased phagocytosis of infected cells and cellular debris and the production of cytokines and chemokines which influence the adaptive immune response (16, 25–30). Activated microglia have been observed in WNV infected *ex vivo* brain (BSC) and spinal cord (SCSC) slice cultures (4, 25). These cultures maintain most of the architecture, cell diversity, and cell-cell interactions of normal brain tissue while isolating microglial responses from peripheral immunity. The use of *ex vivo* slice cultures also eliminates the possibility that PLX5622 may influence peripheral growth and the spread of WNV to the CNS. Previous work using SCSC found that microglia become activated soon after WNV infection and that this process is independent of the peripheral immune response (25).

To further understand the importance of microglia and the role these cells play during WNV infection, the colony-stimulating factor 1 receptor (CSFR1) inhibitor PLX5622 was used to deplete microglia from mice and murine BSC (28, 31). CSF1 signaling is critical for microglia growth and survival (32–34). PLX5622 has been shown to have specificity toward microglia without affecting other cell populations such as brain-specific macrophages, peripheral macrophages, or lymphocytes (28, 35, 36). However, other studies suggest that PLX5622 may affect immune cells in the periphery (27, 37). We showed that depletion of microglia from mice resulted in increased viral loads in the brain and more severe disease in mice infected with WNV (TX02 strain). In addition, we used PLX5622 to deplete microglia from *ex vivo* BSC to specifically investigate the effect of PLX5622 in the brain. Depletion of microglia in WNV-infected BSCs led to increased viral titers and caspase 3 activity, indicating increased cell death in the absence of microglia.

## RESULTS

### Treatment of mice with PLX5622 decreased survival in WNV-infected mice and led to increased viral load in the brain.

**FIG 1:**
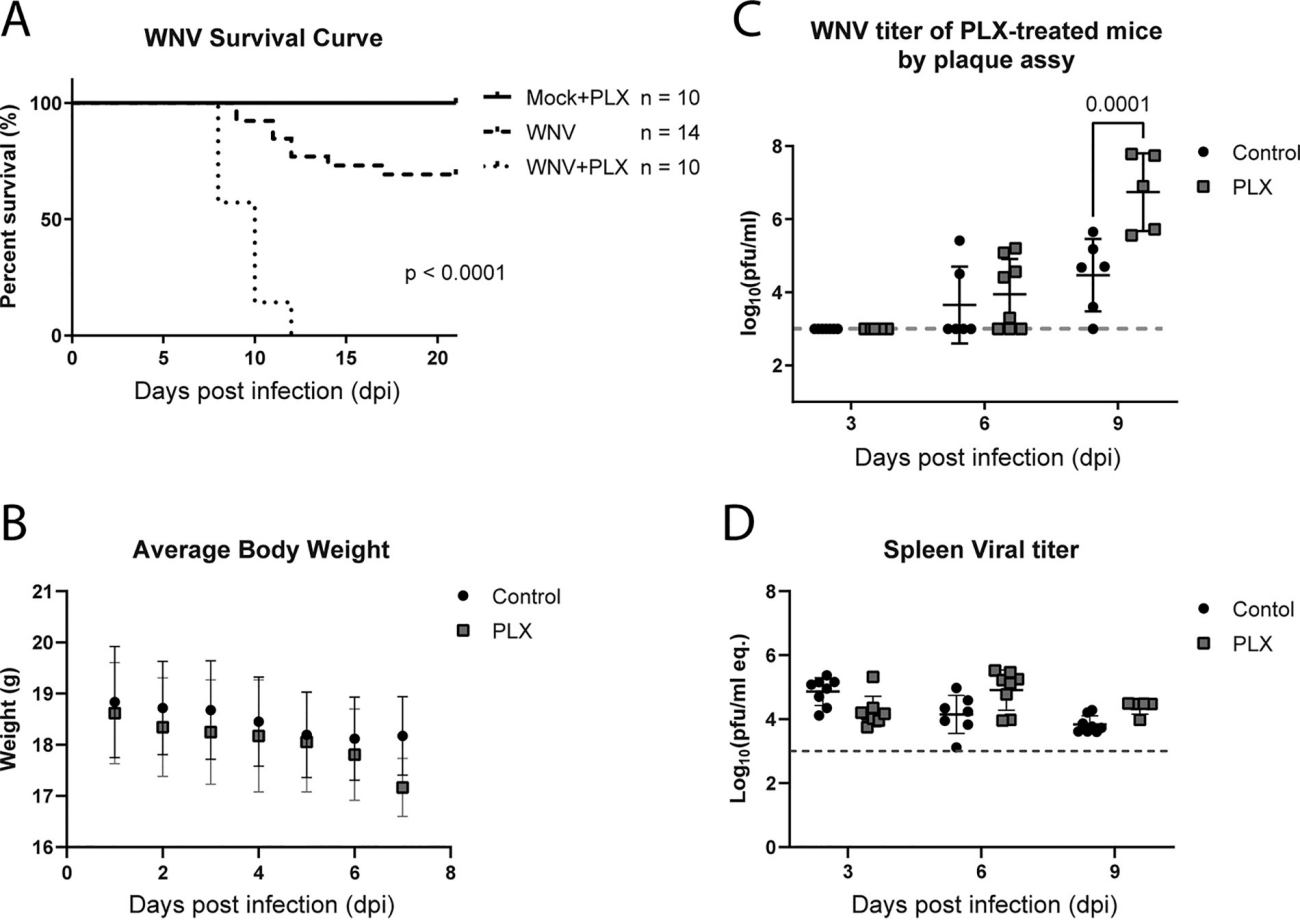
Treatment with PLX5622 decreases survival in WNV-infected mice and increases the viral load in the brain. Mice were fed a diet containing either PLX5622 or control chow for 7 days before infection with 1000 PFU TX02 WNV. Mice were then monitored for 21 dpi. (A) PLX5622 treatment significantly increased mortality in WNV-infected mice with 100% mortality by 12 dpi compared to about 30% mortality by 21 dpi in the control group. Significance was determined by the Gehan-Breslow-Wilcoxon test of survival. (B) Mouse body weights were also recorded throughout the experiment. Viral titers in the brains (C) and spleens (D) of PLX5622 treated and untreated mice were determined by plaque assay and PCR. The graph shows WNV titers in individual PLX5622-treated (gray squares) and untreated (black circles), WNV infected mice. Significance was determined by ANOVA followed by Tukey’s multiple-comparison test using GraphPad Prism 9. The limit of detection for these studies was 1000 and 1300 PFU, respectively. Average titers and SD of the mean are indicated for treated and untreated animals at each time point.

To determine the role of microglia during WNV infection in mice, we depleted the microglia of mice by feeding them a diet containing PLX5622. The efficacy of PLX5622-induced microglial depletion in mice has been established in several previous studies (28, 31). Mice were started on the diet 7 days before infection. The diet was then continued throughout the infection time course. Previous studies using the NY99 strain of WNV found that PLX5622 depletion of microglia before WNV infection led to increased viral titers in the brains of mice. One aim of this study was to repeat this experiment with a more relevant strain of the virus. WNV (strain TX02), which is more similar to the strains currently circulating in the US (38, 39), was more virulent in PLX5622-treated microglia-depleted mice than in the controls (Fig. 1). Mice were infected with 1000 plaque-forming units (PFU) of WNV (TX02) via footpad injection, and weight and survival were monitored daily for 21 days. Weight loss of greater than 20% was used as a humane predictor of subsequent death and animals were sacrificed if this occurred. The PLX5622-treated group showed significantly increased mortality compared to the control treated mice and all mice died between 8 to 12 days postinfection (dpi). In contrast, the control group showed about 70% survival at 21 dpi with mice dying between 9 to 17 dpi (Fig. 1A). Uninfected PLX5622-treated mice presented no morbidity or mortality. There was no significant difference in weight loss between the PLX5622-treated and control-treated groups infected with WNV before 8 dpi when many of the PLX5622-treated animals succumbed to the infection (Fig. 1B). These results suggested that microglia played a critical role in preventing mortality in WNV-infected mice.

Given the robust difference in survival rates seen between the two groups, we next wanted to determine whether PLX5622 affected viral titers within the brains of infected mice. Mice were treated with PLX5622 as described above. At 3, 6, and 9 dpi, brains and spleens (representing peripheral organs) were harvested for analysis of viral titer in both the treated and control groups. Viral titers of harvested brains and spleens were determined using plaque assays and PCR respectively. Mice without detectable virus in the spleen were assumed to have no peripheral infection and were not included in the analysis. Results from mice at 6- and 9-days following infection showed increased viral loads in the brains of PLX5622-treated microglia-depleted mice, compared to control-treated mice (Fig. 1C). There was a significant increase in viral titers in the brains of the mice treated with PLX5622, showing about a 1.5 log increase in the PLX-treated animals compared to the control treatment at 9 dpi. There was no difference in the viral titers in the spleens between the treated and untreated groups (Fig. 1D). These data suggested that *in vivo* PLX5622 treatment in WNV-infected mice led to decreased survival and increased viral titers while peripheral viral titers remained unchanged compared to the control.

### Treatment with PLX5622 depleted microglia in *ex vivo* brain slice cultures.

**FIG 2:**
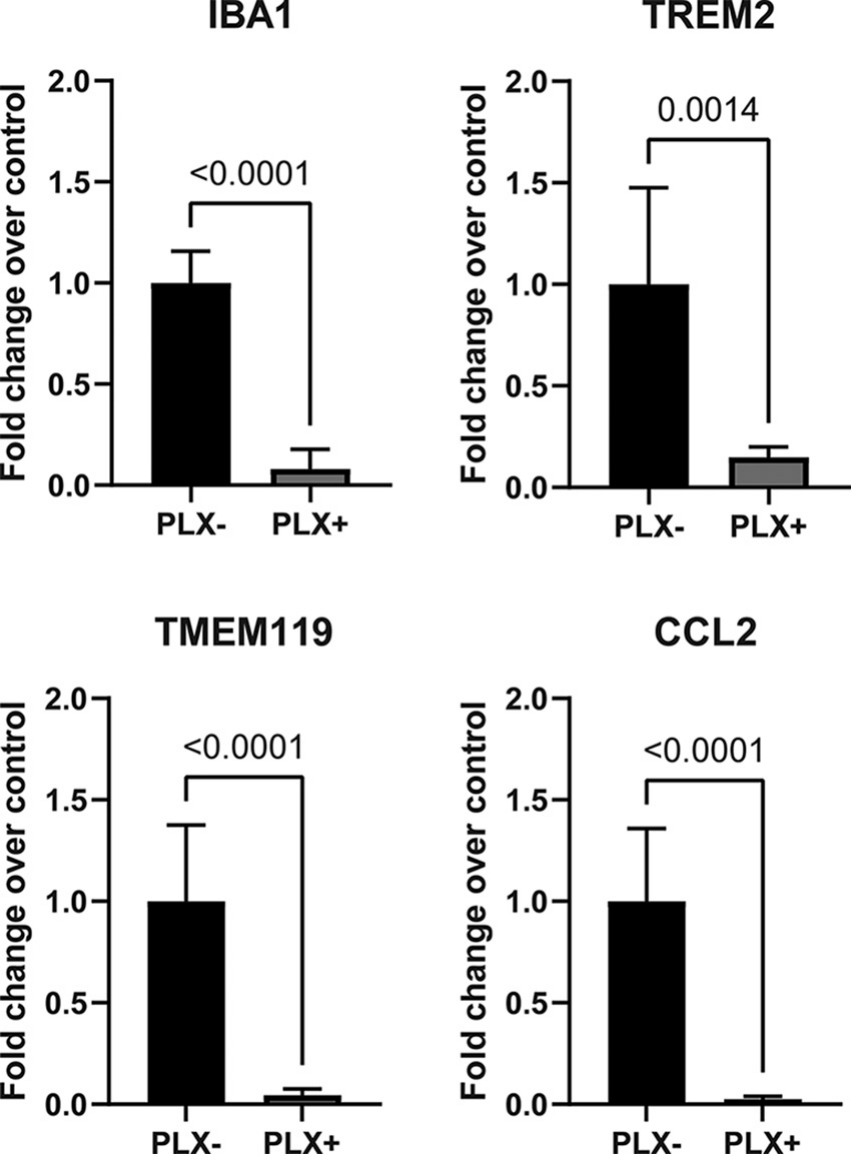
Treatment with PLX5622 depletes microglia in *ex vivo* brain slice cultures. BSCs were treated with 1 μM PLX5622 for 3 days before being collected for microglial gene expression analysis by RT-qPCR. The expression of several microglia-associated genes was determined using RT-qPCR, including IBA1, TMEM119, TREM2, and CCL2. The graph shows the average expression of each gene in treated (gray bars), compared to untreated (black bars) slices. *n* = 5. Error bars are SD of the mean. All genes showed significantly reduced expression in BSCs following treatment with PLX5622 indicating depletion of microglia within the BSCs.

To determine the role of microglia on WNV CNS pathogenesis in isolation from peripheral immune responses, we next determined whether depletion of microglia using PLX5622 could be accomplished in a murine *ex vivo* BSC model. BSCs were cut from the brains of 4-day-old Swiss Webster mouse pups and placed on a culture membrane insert. Slices were treated with 1 μM PLX5622 for 3 days before being harvested for RNA preparation and gene expression analysis. The quantification of multiple microglia-associated genes was determined using RT-qPCR. These genes included Iba1 (ionized calcium-binding adaptor molecule 1), TMEM119 (transmembrane protein 119), TREM2 (triggering receptors expressed on myeloid cells 2), and CCL2 (C-C motif chemokine ligand 2), which are commonly used markers for measuring the activation and/or presence of microglia (37–39). Iba1 is a surface molecule specific to microglia and plays a role in regulating the activation of microglia and is, thus, commonly used as a proxy for measuring the activation and presence of microglia (40). CCL2 is a potent microglial cytokine and shows increased expression following activation of microglia in the context of neuroinflammatory diseases (41). As expected, compared to vehicle (DMSO)-treated controls, the expression of Iba1, TMEM119, TREM2, and CCL2 were reduced in PLX5622 treated slices (Fig. 2). The significant reduction in the expression of these microglia-associated genes revealed that PLX5622 was effective at depleting microglia in *ex vivo* brain slice cultures. We next used this model of BSC microglia depletion to investigate the effect of PLX5622 on the outcome of infection with WNV.

### PLX5622 treatment led to increased viral titers and caspase 3-mediated apoptosis in WNV-infected brain slice cultures.

**FIG 3:**
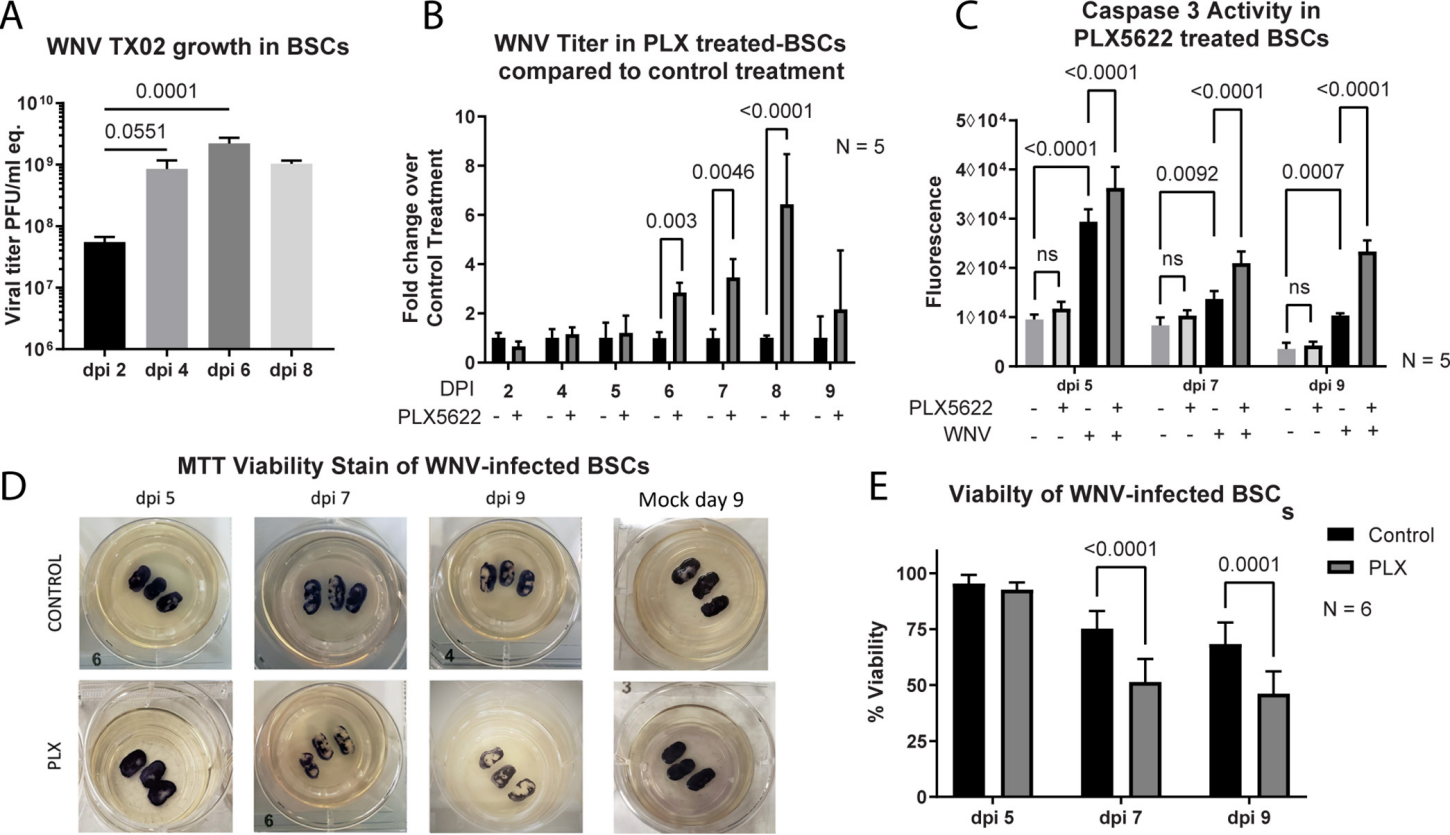
PLX5622 treatment in WNV-infected BSCs leads to increased viral titers and caspase 3 mediated apoptosis. (A) BSCs were infected with 10^5^ PFU of WNV (TX02) and samples were harvested at 2, 4, 6, and 8 days postinfection for determination of viral titer by PCR. (B) BSCs were treated with either PLX5622 or the vehicle control and infected with 10^5^ PFU/mL of WNV (TX02). At the indicated times, cultures were harvested, and the titer was determined using RT-PCR. For each day, data were normalized to control treatment (set to 1) and any increase seen in the PLX5622 treated samples indicated a fold increase in viral titer. The graph shows the mean titer. Error bars are SD. (C) Caspase 3 activity in lysates from WNV- and mock-infected PLX5622-treated and untreated BSCs was determined using a fluorometric caspase 3 assay. The graph shows the mean titer. Error bars are SD. (D) MTT staining of BSCs from each treatment group at 5, 7, and 9 dpi, as well as mock samples from 9 dpi. The purple tissue in the pictures represents viable cells while the white tissue is dead. (E) The percent viability is shown in the bar graph with error bars representing SD.

Having established that PLX5622 could deplete microglia in BSC, we then sought to determine whether the loss of microglia influenced viral titers in WNV-infected BSC. We first demonstrated that WNV (TX02) grew in BSC. BSCs were prepared (42) and infected with 10^5^ PFU/well of the TX02 WNV strain. Slices were harvested at 2, 4, 6, and 8 dpi, and viral titers were determined by PCR. By day 2, there was already an almost 1000-fold increase in viral titer, which increased to a more than 10,000-fold increase in titer by day 6 before beginning to decline by day 8 (Fig. 3A). The decline was likely due to the BSCs losing viability due to the virus-induced cytopathic effects. The peak in viral titer was seen at 6 dpi, at which point the titer reached 10^9^ PFU/mL. These data confirmed that WNV (TX02) could infect and grow in *ex vivo* BSC.

To determine the effect of microglia depletion on viral titers in WNV-infected BSC, cultures were treated with either PLX5622 or the control vehicle (DMSO) for 3 days before infection to achieve microglia depletion. Treatment was then continued throughout the time course of the infection along with daily media changes. BSCs were infected with 10^5^ PFU of the TX02 WNV strain and were harvested at 2, 4, 5, 6, 7, 8, and 9 dpi. Each data point was normalized to its corresponding control treatment to mitigate variability between experiments. Compared to control treatment, viral titers were significantly increased in the PLX5622 treated BSCs on days 6, 7, and 8 postinfection with 8 dpi showing the greatest increase at approximately 6 times higher than the control treatment (Fig. 3B). Viral titers between the two treatment groups were similar at days 1 to 5 postinfection. This suggested that microglia were critical for the eventual control of WNV infection and were particularly important at late times following infection.

Once inside the CNS, WNV can directly infect neurons and induces cell death by caspase 3-dependent apoptosis (4, 5). Given that PLX5622 treatment led to increased viral titer in the BSCs, we examined whether WNV-induced neuronal cell death by caspase 3 apoptosis was also increased in the absence of microglia. The same samples used to obtain viral titers at 5, 7, and 9 dpi were used in fluorometric caspase 3 activity assays to determine the amount of caspase 3 activity within the PLX5622 and control-treated BSCs following WNV infection. At all three time points, caspase 3 activity was increased in the PLX-treated BSCs compared to the control treatment (Fig. 3C). Also, PLX5622 alone did not significantly increase caspase 3 activity in mock-infected BSCs. These results suggested that microglia have a role in limiting caspase 3 apoptotic cell death during a neuroinvasive WNV infection.

To further investigate the effect of microglia depletion on cell death, WNV-infected BSCs treated with PLX5622 were stained using MTT (3-[4,5-dimethylthiazol-2-yl]-2,5-diphenyltetrazolium bromide) to measure tissue viability (43). In the WNV infected BSCs, PLX5622 treatment led to a decrease in viability by 7 dpi compared to the control treatment. While both groups lost viability as the infection progressed, microglia depletion led to a 15% to 20% greater decrease in viability compared to the corresponding controls (Fig. 3D and E). This suggested that the increase in caspase 3 activity seen in the PLX5622 treated BSCs (Fig. 3B) resulted in reduced cell viability of the microglia-depleted BSCs following WNV infection.

### PLX5622 did not block WNV-induced expression of proinflammatory and anti-inflammatory cytokines or genes involved in interferon (IFN) signaling.

**FIG 4:**
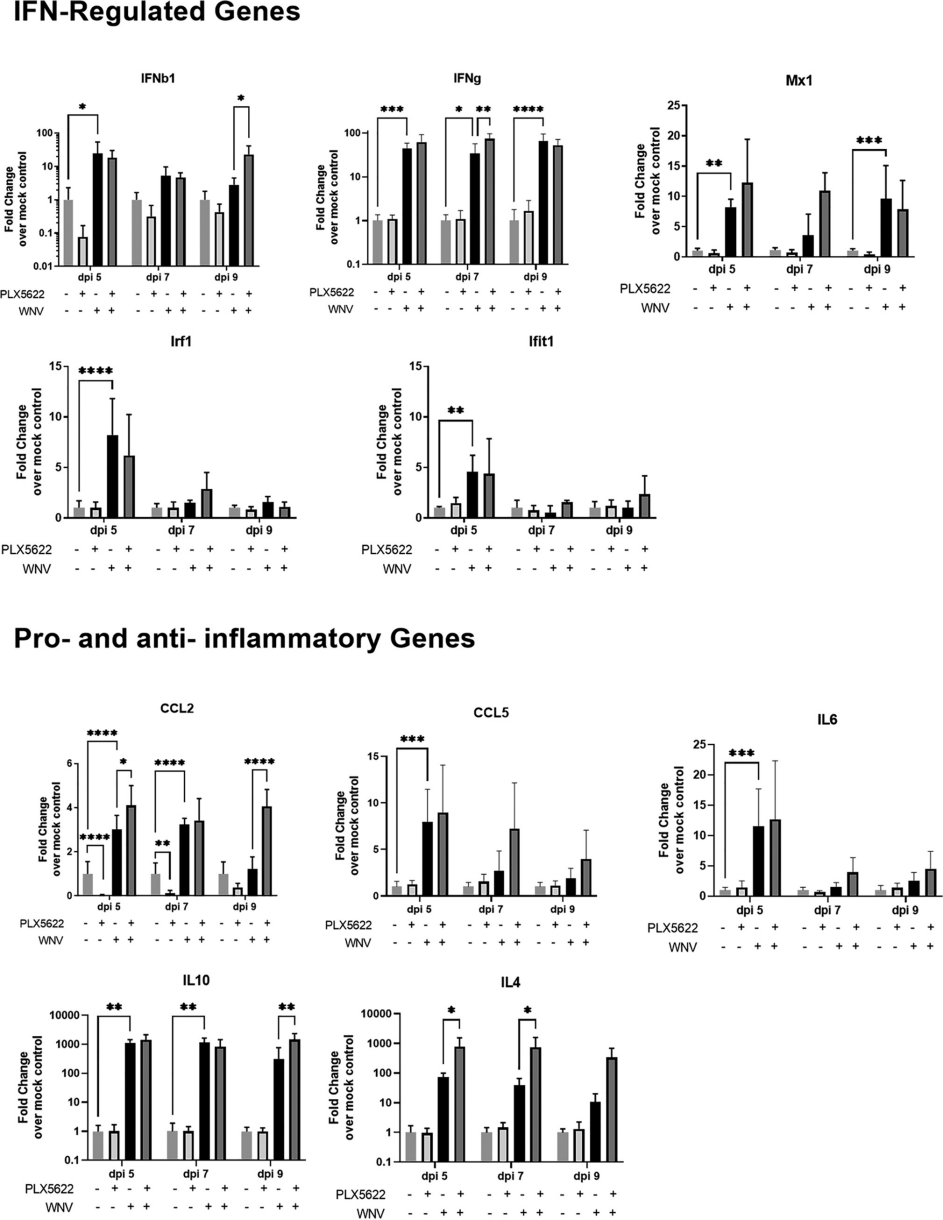
PLX5622 does not block WNV-induced expression of proinflammatory and anti-inflammatory cytokines or genes involved in interferon (IFN) signaling. BSCs were infected with TX02 WNV and treated with either PLX or the control and then harvested for analysis on days 5, 7, and 9 postinfection. Several cytokines known to be involved in the microglial response to WNV were analyzed for relative expression. Graphs show relative gene expression normalized to the control-treated mock-infected samples. The comparisons of interest are as follows: PLX-WNV- versus PLX^+^WNV^−^; PLX^−^WNV^−^ versus PLX^−^WNV^+^; WNV^+^PLX^−^ versus WNV^+^PLX^+^. Only significant differences between these groups are shown on the graph and these are represented with asterisks (*, *P* ≤ 0.05; **, *P* ≤ 0.01; ***, *P* ≤ 0.001; ****, *P* ≤ 0.0001). Statistical significance was determined by ANOVA followed by Tukey’s multiple comparisons. *n* = 5. Error bars represent the standard deviation from the mean.

One function of microglia is the production of cytokines and chemokines, which can alter inflammatory processes and recruit lymphocytes to the CNS. We next wanted to determine whether PLX5622 depletion of microglia had an impact on the expression levels of cytokines and chemokines in WNV-infected BSCs. This was done by determining the expression of these genes using RT-qPCR in lysates of treated/infected BSCs (Fig. 4). Specific cytokines and chemokines that were triggered following WNV infection and known to influence inflammation (proinflammatory and anti-inflammatory genes), recruitment of lymphocytes, and IFN signaling were chosen for this role (44–46), including CCL2, CCL5, interleukin (IL)-6, IL-4, IL-10, IFN-β1, IFN-γ, MX1, IFN regulatory factor 1 (IRF1), and IFN-induced protein with tetratricopeptide repeats 1 (IFIT1). Out of all the genes investigated only CCL2 showed decreased expression in PLX5622-treated, uninfected BSC compared with untreated controls, as we have previously shown (Fig. 2). Depletion of microglia did not decrease the expression of CCL5, IL-4, IL-6, and IL-10 in uninfected BSC, suggesting that other cell types regulate the expression of these genes. As expected, the expression of genes involved in the antiviral IFN response to WNV infection was also not altered in PLX5622-treated, uninfected cells.

Following WNV-infection of BSC, there was a widespread increase in the expression of proinflammatory and anti-inflammatory cytokines along with genes involved in IFN signaling (Fig. 4) as previously reported (25, 44, 45). This increased expression was maintained (if not enhanced) in the absence of microglia following PLX5622 treatment, suggesting microglia were not required for the WNV-induced expression of these cytokines. CCL2, IFN-γ, IL-4, and IL-10 all showed increased expression in the WNV-infected cultures following PLX5622 treatment. This is likely a result of a heightened response from other cells to the enhanced viral growth in the absence of microglia. Although microglia were important for CCL2 expression in uninfected cells, the WNV-induced increase in CCL2 and other cytokines expression did not appear to be driven by microglia.

### Astrocytes showed increased activation following WNV infection in microglia-depleted BSCs.

To address whether the increase seen in cytokine expression in PLX-treated BSCs following WNV infection could be due to a heightened response from astrocytes, we determined the expression of several astrocyte-related genes. These genes included GFAP (glial fibrillary acidic protein), VIM (vimentin), LCN2 (lipocalin 2), and STEAP4 (six transmembrane epithelial antigens of prostate 4). GFAP is used as a marker for astrocytes to distinguish these cells from other glia (47). VIM encodes a type III intermediate filament protein and can also be used as a marker for astrocytes (48, 49). LCN2 encodes a protein that is involved in the transport of small molecules within the cell and is upregulated in astrocytes in inflammatory environments (50, 51). Lastly, STEAP4 encodes a metalloreductase that functions in the Golgi apparatus of the cell, and this gene acts as a transcriptionally regulated marker of astrocytic activation (52, 53).

**FIG 5:**
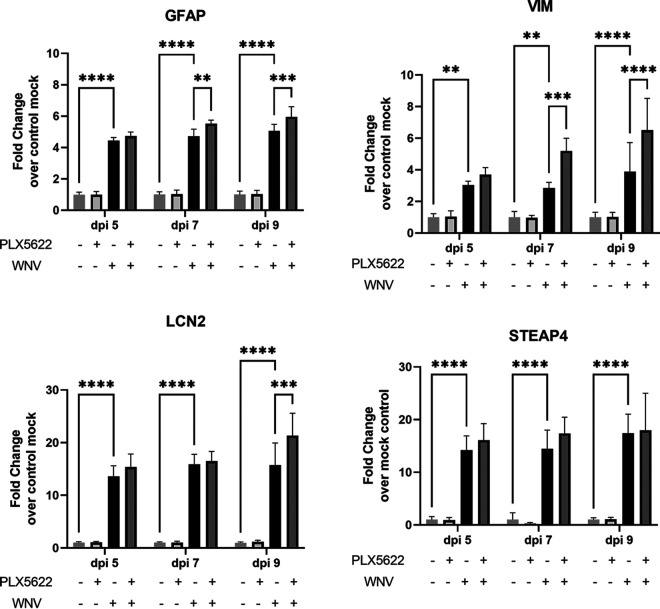
Increased expression of astrocyte-related genes in BSCs following infection with WNV and microglia depletion with PLX5622. BSCs were infected with TX02 WNV and treated with either PLX or the control and then harvested for analysis on days 5, 7, and 9 postinfection. Several cytokines involved in astrocyte activation were analyzed for relative expression to the control-treated mock samples. The comparisons of interest are as follows: PLX^−^WNV^−^ versus PLX^+^WNV^−^; PLX^−^WNV^−^ versus PLX^−^WNV^+^; WNV^+^PLX^−^ versus WNV^+^PLX^+^. Only significant differences between these groups are shown on the graph and these are represented with asterisks (*, *P* ≤ 0.05; **, *P* ≤ 0.01; ***, *P* ≤ 0.001; ****, *P* ≤ 0.0001). Statistical significance was determined by ANOVA followed by Tukey’s multiple-comparison test. *n* = 5. Error bars represent the standard deviation from the mean.

Activation of astrocytes is expected to occur in the presence of WNV (45), and this was observed in the BSCs following WNV infection because GFAP, VIM, LCN2, and STEAP4 all showed enhanced expression following infection with WNV compared with the mock-infected samples (Fig. 5). GFAP and VIM showed about a 3 to 4-fold increase in expression by day 5 postinfection, and LCN2 and STEAP4 showed a greater than 10-fold increase in expression by day 5 postinfection (Fig. 5). Further analysis of PLX5622 treated samples revealed an even higher expression of some of these genes following WNV infection. Of the genes listed above, GFAP, VIM, and LCN2 showed increased expression in the PLX5622 treated brain slice cultures following WNV infection compared to the control-treated group (Fig. 5). These results supported our hypothesis that astrocytes may be responsible for the heightened cytokine production in the PLX-treated samples. This effect was seen mostly at the later time points of the experiment, indicating that the increased viral titers seen in the PLX5622 treated BSCs by day 6 postinfection may be leading to enhanced activation of astrocytes.

## DISCUSSION

Microglia play a major role in the innate immunity of the CNS along with several other vital functions in neuronal development and homeostasis. This study aimed to better define the role of microglia during neuroinvasive WNV infection. We first showed that survival of mice infected with the TX02 stain of WNV was greatly reduced following PLX5622 treatment, with 100% of mice succumbing to infection between 8 to 12 dpi in the treated group, compared to 30% mortality occurring between 9 and 17 dpi in untreated control animals. Decreased survival was associated with increased viral titers in the brain (Fig. 1), indicating the cause for increased mortality may be overwhelming infection within the CNS in the absence of microglia. These results are consistent with similar studies showing that PLX5622 increases disease severity following infection of mice with human coronavirus, Japanese encephalitis virus, and other noncirculating strains of WNV (28, 31).

Increased viral titer in the brain following PLX5622 treatment could be a direct result of microglial depletion because microglia likely contributes to viral clearance by phagocytosing infected cells (25) and by influencing immune responses (45). Alternatively, PLX5622 may affect peripheral immunity resulting in higher peripheral titers that allow more virus to access the CNS (27). Our studies indicated that viral replication in the periphery was not affected by PLX5622. However, a previous report using a different WNV strain suggested otherwise (27). To further examine the role of PLX5622 in the CNS in isolation from peripheral effects, either viral or immune-related, we used an *ex vivo* brain slice culture model of WNV pathogenesis.

Ex vivo BSCs maintain much of the diverse architecture and cell types within the brain while also removing any effects on the peripheral immune system. To confirm that PLX5622 could deplete microglia in the BSCs we demonstrated the decreased expression of microglial markers (IBA1, TMEM119, TREM2, and CCL2). The expression of all these genes was significantly reduced following PLX5622 treatment providing strong evidence for the efficient depletion of microglia in this BSC model. This supports a previous study that showed that PLX5622 can selectively deplete microglia in BSCs without affecting other cell populations (54). PLX5622-treated BSC was then used to demonstrate that depletion of microglia results in increased viral titer and caspase 3 activations following WNV infection as well as decreased BSC viability.

PLX5622 treated BSCs showed significantly increased viral titers when normalized to the control treatment at 6, 7, and 8 dpi while showing no difference in viral titers during the earlier days of infection. This suggests that microglia are important a few days following initial infection and that they function to limit viral growth. The exact mechanism by which microglia control WNV growth remains unclear; however, due to their phagocytic phenotype in WNV infected BSC (25) it is likely that phagocytosis of viral particles and virus-infected neurons contributes to this ability.

Along with limiting viral growth, microglia were also found to contribute to preventing caspase 3 mediated apoptotic cell death and loss of tissue viability (Fig. 3). This could be a direct effect of reducing viral load within the BSCs or an indirect effect, such as modulation of inflammation. However, the former seems more likely given that PLX5622 depletion of microglia had little effect on many inflammatory cytokines/chemokines, which show increased expression during a neuroinvasive WNV infection (Fig. 4). Limiting viral growth in the BSCs would lead to fewer WNV-infected neurons and thus less WNV-induced apoptosis via caspase 3 activation. These data suggest that microglia aid in protection from neuroinvasive WNV infections by limiting viral growth and thus subsequent virus-induced caspase 3 apoptosis of neurons in the absence of infiltrating peripheral immune cells.

The lack of differences in cytokine expression, which is contradictory to what was expected in a microglia-depleted environment, is most likely due to other cells within the CNS compensating for the loss of microglia and is consistent with studies investigating microglial depletion in an *in vivo* model of WNV pathogenesis (32). The loss of microglial control of viral growth seems to induce stronger virus-induced cytokine production in non-microglia cells. This same result had been observed previously in other studies investigating the effects of PLX-mediated depletion of microglia on inflammatory cytokine production (31, 36). One cell type which may be responsible for the increase in cytokines in the PLX5622-treated WNV infected samples is the astrocyte population within the brain. Astrocytes perform many roles within the CNS, including providing support to the cells, which form the blood-brain barrier, maintaining extracellular homeostasis, and provision of nutrients to neurons (55). These cells have been shown to become activated following WNV infection (45), and, thus, we investigated whether there is increased expression of astrocyte-related genes following treatment with PLX5622. Several genes involved in astrocyte activation showed increased expression, including GFAP, VIM, and LCN2 and this increased expression in the PLX5622 treated group occurred during the later time points of between 7 and 9 days postinfection. This provides evidence that astrocytes may be at least partly responsible for the increase in the expression of certain cytokines following depletion of microglia in WNV infected BSCs and that they become activated when viral burden increases.

Microglia perform both proinflammatory and anti-inflammatory roles during viral infections (18, 46). There has been a significant amount of research into whether the proinflammatory or anti-inflammatory roles of microglia are more essential in protection from viral infections. One such study used the drug minocycline to induce an anti-neuroinflammatory environment in spinal cord slice cultures following WNV infection. Treatment was found to reduce cytotoxicity in the spinal cord slice cultures indicating that the inflammatory effects of microglia may be harmful to neuronal health (26). Anti-inflammatory effects may thus improve long-term outcomes of WNV infections. In contrast, another recent study used the drug rosiglitazone to inhibit neuroinflammation and found similar results to those reported here. Rosiglitazone treatment of WNV-infected murine brain slice cultures led to increased viral titers and caspase 3 activations (30). These studies bring into focus the complex nature of microglia and neuroinflammation and more studies are needed to fully understand the role of microglia and neuroinflammation in the defense from WNV infections.

The impact of microglial activity during a neuroinvasive WNV infection is complicated and may change throughout the infection. Several studies have shown the importance of microglia during the initial stages of viral infections (28, 31). However, other studies have shown that microglia may contribute to neurodegeneration during the late stages of WNV infections (29, 56). A recent study by Garber et al. (56) revealed that memory T cells promote synapse loss by microglia following clearance of the WNV infection leading to neurodegenerative processes. Another study by Vasek et al. (29) showed that the complement system is involved in driving microglial-mediated synapse loss.

Our previous results suggest that microglia directly limit viral growth through a phagocytic process, likely phagocytosis of viral particles or virus-infected neurons because the loss of microglia does not have an impact on the production of cytokines/chemokines which would limit viral growth indirectly through inflammatory processes and the recruitment of peripheral lymphocytes. The data presented in this paper supported previous studies (28, 31), emphasizing the importance of microglia in the pathology of viral infections and demonstrating that microglia are protective in the absence of peripheral immune responses.

## MATERIALS AND METHODS

### Mice.

All experiments were performed with approval from the Institutional Animal Care and Use Committee (IACUC). Eight-week-old female C57BL/6 mice were used for the *in vivo* portion of this study. Our previous studies have shown no significant difference in lethality between female and male mice. Mice were monitored daily, and body weights were obtained and cataloged per protocol.

### Preparation of murine brain slice cultures.

Pregnant Swiss Webster mice were obtained, and the time of litter birth was recorded. Four-day-old Swiss Webster pups were decapitated, and brains were harvested per protocol. Following removal of the cerebellum, brains are mounted on the cutting disk with a small amount of HDX super glue. The disk was then secured to the Vibratome (Leica VT1000S) and submerged under cold DMEM + 1% HEPES. Following removal of about the first 800 μm of tissue to eliminate the olfactory bulb, 3 subsequent 400 μm thick slices are prepared for culture on Millicell cell culture inserts.

### PLX5622 preparation and treatment.

PLX5622 was provided by Plexxikon for the experiments presented in this study. Research Diets prepared the PLX5622 Chow on the AIN-76A high sucrose formulation. For the *in vivo* experiments, mice were fed either the PLX5622 or control chow for 7 days before infection and the diet was maintained throughout the 21 days of infection. For the *ex vivo* brain slice cultures, 10 μM stocks were prepared in DMSO, and slices were treated with 1 μM PLX5622 (54).

### WNV stocks and inoculation.

The TX02 stain of WNV was prepared by passaging the virus through C6/36 mosquito cells to amplify the virus. Once cytopathic effects were observed, the virus was purified by sucrose ultracentrifugation and diluted to predetermined inoculums in sterile phosphate-buffered saline (PBS). The inoculation of the 8-week-old female C57BL/6 mice used in the *in vivo* portion of this study involved injection of 1000 PFU of the virus strain into the left rear footpad. Brian slice cultures were inoculated with 10^5^ PFU/well of the TX02 strain.

### RNA isolation and reverse transcription-quantitative PCR (RT-qPCR).

Homogenates were prepared from either whole brains or brain slice cultures in 1 mL of PBS using a Bead bug homogenizer. Following homogenization, 100 μL of homogenate was added to 200 μL of RLT buffer. RNA was then isolated from this solution using Qiagen RNeasy Minikit following the manufactures instructions. RNA was converted to cDNA using the Qiagen iScript kit. RT-qPCR was performed on a Bio-Rad CFX1000 instrument and analyzed using Bio-Rad software before statistical analysis using GraphPad Prism 9 Software. Unpaired *t* tests and ANOVAs followed by Tukey's multiple-comparison test were used to determine statistical significance. Data were normalized to mock control-treated samples to eliminate variability between experiments. Error bars are representative of the standard deviation from the mean.

### Determination of WNV titer.

WNV titers were determined by both plaque assay and PCR. Plaque assays were performed using VERO cells and homogenates prepared from mouse brains to quantify the amount of virus within the brain. WNV titers were also determined by PCR using the RNA isolated from mouse brains as described above. Viral titers were transformed to their respective Log_10_ values for analysis. Any mice which had no detectable virus in peripheral organs or in the brain were excluded from the analysis. In total, 3 mice were found to be uninfected and thus were not included.

### Caspase 3 activity assay.

A fluorometric caspase 3 assay was used to determine the level of caspase 3 activations within samples. Samples were prepared following the manufacturer’s instructions. A Cytofluor 4000 spectrometer plate reader was used to determine fluorescence at an excitation wavelength of 450 nm and an emission wavelength of 530 nm. Statistical analysis was done by ANOVAs followed by Tukey's multiple-comparison test using GraphPad Prism 9 software and error bars represent standard deviation from the mean.

### MTT viability stain.

MTT (3-[4,5-Dimethylthiazol-2-yl]-2,5-Diphenyltetrazolium bromide) was used to determine cell viability as a function of redox potential. Living cells will convert MTT to purple formazan. To stain the brain slice cultures, 100 μL of the MTT solution was applied to the BSCs and allowed to incubate for 30 min at 37°C before viewing. Pictures were taken of the stained slices and ImageJ was used to determine the percentage of viable tissue. Statistical analysis by ANOVA was performed using GraphPad Prism 9 software. Error bars are representative of the standard deviation from the mean.
